# An active electrode for biopotential recording from small localized bio-sources

**DOI:** 10.1186/1475-925X-3-25

**Published:** 2004-07-22

**Authors:** Emil S Valchinov, Nicolas E Pallikarakis

**Affiliations:** 1 Department of Medical Physics, University of Patras, Patras 26500, Greece

## Abstract

**Background:**

Laser bio-stimulation is a well-established procedure in Medical Acupuncture. Nevertheless there is still a confusion as to whether it works or the effect is just placebo. Although a plethora of scientific papers published, showing positive clinical results, there is still a lack of objective scientific proofs about the bio-stimulation effect of lasers used in Acupuncture. The objective of this work was to design and build a body surface electrode and an amplifier for biopotential recording from acupuncture points, considered here as small localized bio-sources (SLB). The design is aimed for studying SLB potentials provoked by laser stimulus, in search for objective proofs of the bio-stimulation effect of lasers used in Medical Acupuncture.

**Methods:**

The active electrode presented features a new adjustable anchoring system and fractionation of the biopotential amplifier between the electrode and the cabinet's location. The new adjustable electrode anchoring system is designed to reduce the electrode-skin contact impedance, its variation and motion artifacts. That is achieved by increasing the electrode-skin tension and decreasing its relative movement. Additionally the sensing element provides local constant skin stretching thus eliminating the contribution of the skin potential artifact. The electrode is attached to the skin by a double-sided adhesive pad, where the sensing element is a stainless steel, 4 mm in diameter. The fractionation of the biopotential amplifier is done by incorporating the amplifier's front-end op-amps at the electrodes, thus avoiding the use of extra buffers. The biopotential amplifier features two selectable modes of operation: semi-AC-mode with a -3 dB bandwidth of 0.32–1000 Hz and AC-mode with a bandwidth of 0.16–1000 Hz.

**Results:**

The average measured DC electrode-skin contact impedance of the proposed electrode was 450 kΩ, with electrode tension of 0.3 kg/cm^2 ^on an unprepared skin of the inner forearm. The peak-to-peak noise voltage measured at the amplifier output, with input terminals connected to common, was 10 mV_p-p_, or 2 μV_p-p _referred to the input. The common-mode rejection ratio of the amplifier was 96 dB at 50 Hz, measured with imbalanced electrodes' impedances. The prototype was also tested practically and sample records were obtained after a low intensity SLB laser stimulation. All measurements showed almost a complete absence of 50 Hz interference, although no electrolyte gel or skin preparation was applied.

**Conclusion:**

The results showed that the new active electrode presented significantly reduced the electrode-skin impedance, its variation and motion artifact influences. This allowed SLB signals with relatively high quality to be recorded without skin preparation. The design offers low noise and major reduction in parts, size and power consumption. The active electrode specifications were found to be better or at least comparable to those of other existing designs.

## Background

The non-invasive nature of laser bio-stimulation have made lasers an attractive alternative in Medical Acupuncture at the last 25 years. Unfortunately there is still a confusion as to whether they work or their effect is just placebo. Although a plethora of scientific papers published, showing positive clinical results, there is still a lack of objective scientific proofs about the bio-stimulation effect of lasers used in Acupuncture. The objective of this work was to design and built a body surface electrode and an amplifier for biopotential recording from acupuncture points. The latter are considered here as small localized bio-sources (SLB). As discussed by other authors, SLB are small area body regions with specific electrical, physiological and anatomical properties (e.g. high density of gap junctions, relatively low impedance etc.) [1-4]. They appear to be highly sensitive to mechanical, thermal, electrical or electromagnetic stimulation and are found to take place from the epidermis (stratum granulosum) to a maximum depth of 2 cm [5-8].

The active electrode is aimed for studying SLB potentials provoked by laser stimulus, in search for objective proofs of the bio-stimulation effect of lasers used in Medical Acupuncture.

## Methods

### Electrode design

The attempt to define the optimal parameters of the active electrode was based on a set of preliminary measurements performed in our laboratory. Anatomical, physiological and electrical characteristics of the signal source were considered. The SLB AC signal level, after stimulation, varied from subject to subject, but did not exceed 1 mV peak-to-peak (p-p). Additionally SLB occasionally manifested a high DC potential up to 200 mV, implying the use of differential amplifier with optional DC coupling and wide DC input range. The frequency band of the signal of interest was found to be in the range 0–200 Hz. The preliminary experiments showed that SLB potentials were best recorded with small pasteless electrodes although their contact impedance depends strongly on sweat gland secretion. The application of electrolytic gel resulted in significant reduction of the SLB signal amplitude, probably due to smoothing of the potential caused by saturation of the epidermis with electrolyte. Moreover, potentials between closely spaced SLB might be shortened by the application of excessive gel or large surface electrodes. An additional difficulty is that the SLB are often situated at convex or concave body surface areas where large flat electrodes could not be easily affixed. Skin abrasion with sandpaper is also not recommended since it can cause skin irritation and SLB potential changes. However, the use of small passive dry electrodes on an unprepared skin results in high electrode-skin impedance, motion artifacts, high power-line cable interference and noise. When the electrodes are DC coupled to the amplifier, a motion induced interfering signal appears at the amplifier input, mainly due to:

• Electrokinetic effect – the disturbance of the double layer of charge at the electrode-skin interface causes variations of the DC polarization potential [9].

• Skin potential or skin stretch artifact – stretching of the skin causes a change of the potential of the barrier layer between the epidermis and the dermis [10].

• Variation in the electrode-skin contact resistance – caused by the amplifier input bias current and the current flowing due to the polarization potential.

**Figure 1:**
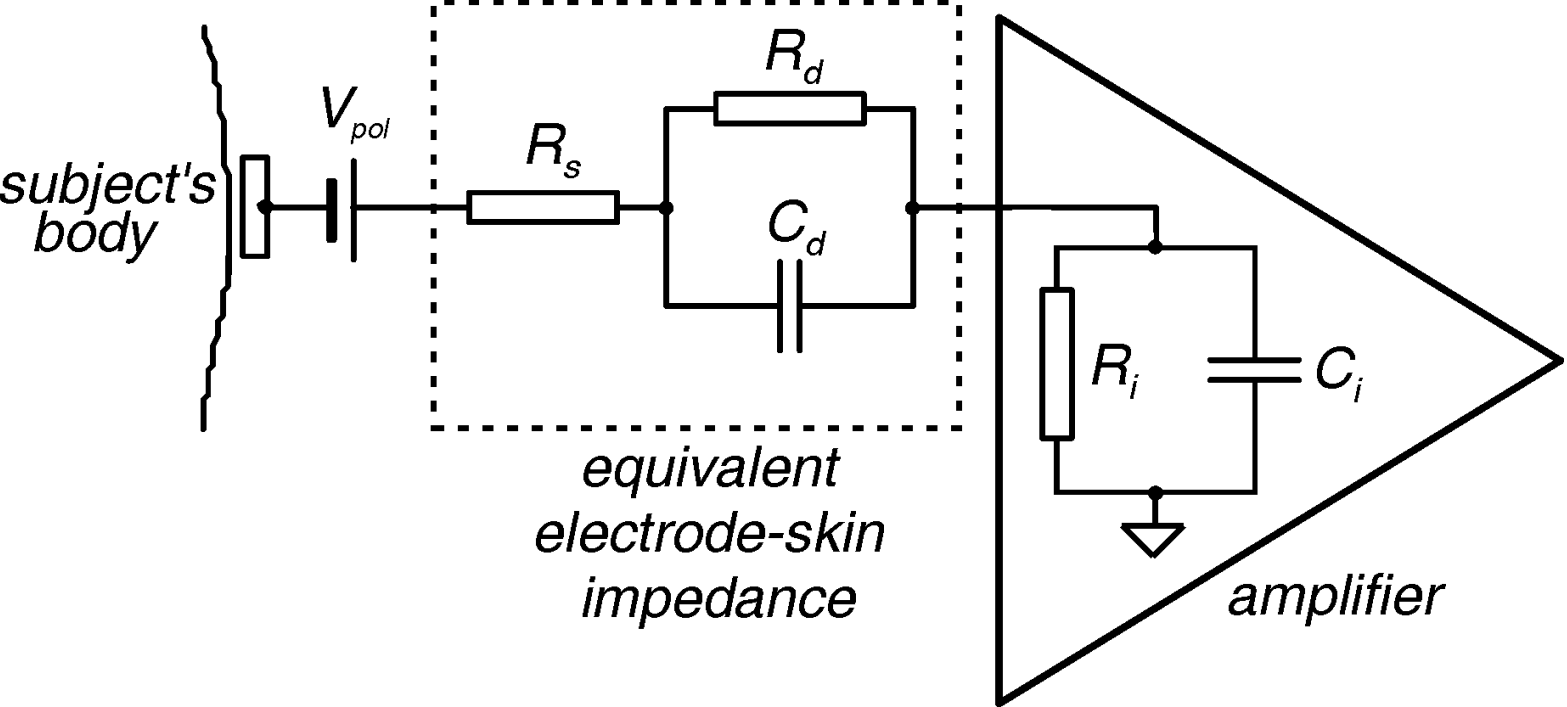
Equivalent electrical model of the electrode-skin interface.

The complex electrochemical interactions that take place at the electrode-skin interface have been subject to much study in order an equivalent electrical model to be developed [10-12]. The simple but adequate electrical model used, is shown in Fig. 1, where C_d_//R_d _is the coupling impedance of the double layer at the electrode-skin interface, C_i_//R_i _is the amplifier input impedance, R_s _is the minimum series contact resistance and V_pol _is the DC polarization potential. Then the motion artifact signal at the amplifier input can be expressed as

*V*_*mot *_= Δ*V*_*pol *_+ Δ*V*_*skin *_+ (Δ*R*_*d *_+ Δ*R*_*s*_) (*V*_*POL *_/ *R*_*i *_+ *i*_*b*_)     (1)

where ΔV_skin _is the skin stretch artifact and i_b _is the amplifier input bias current. It was deduced that in order to keep the resistive interfering component less than 10 μV when DC coupling is employed and with both currents contributing equally to it, then i_b_<50 pA and R_i_>1 GΩ [11]. If an AC coupling is used then the resistive component of the motion artifact is eliminated.

**Figure 2:**
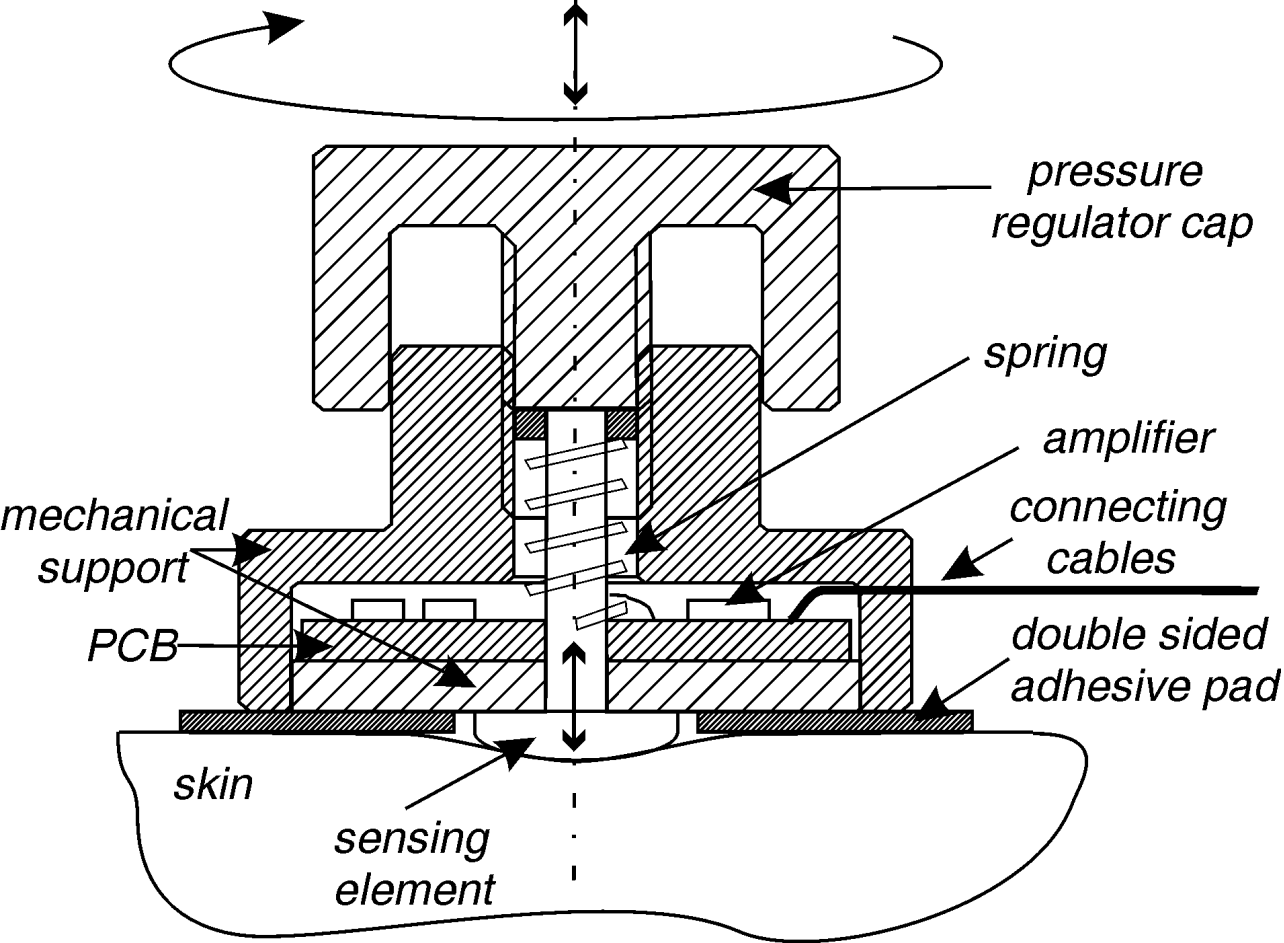
Dry active electrode with adjustable anchoring system.

For dry electrodes the motion artifacts are mainly caused by changes of the polarization potential and the contact impedance due to the poor electrolyte layer at the electrode-skin interface. Therefore a firm electrode-skin contact is of primary importance. Thus a new adjustable electrode anchoring system was designed for the purpose, as shown in Fig. 2. Turning the electrode cap clockwise pushes the sensing element against the skin. Thus electrode-skin pressure is increased, leading to reduction of the contact impedance and its variation. The electrode-skin relative movement is also reduced, making the noise contribution of the electrokinetic effect insignificant. Additionally, the sensing element provides constant skin stretching that lowers the contribution of the skin potential artifact. Turning the electrode cap counter-clockwise, releases the spring, which pushes back the sensing element, resulting in a lower electrode-skin pressure. Titanium, stainless steel and aluminum were considered as electrode sensing materials. Stainless steel was chosen because it is more commonly available than titanium and preferable to aluminum, which has been shown to have problems due to chemical response of its oxide to perspiration [13]. The use of stainless steel electrode material ensures low noise, minimal offset potentials and excellent DC stability suitable for low-drift DC measurements [14]. The sensing element is a 4 mm diameter, selected according to the average SLB size. Although the obtained contact impedances with the prototype electrode were relatively low for dry electrodes of that size, impedance matching at the electrode site was still needed to cope with the power-line cable interference.

### Basic amplifier circuit

**Figure 3:**
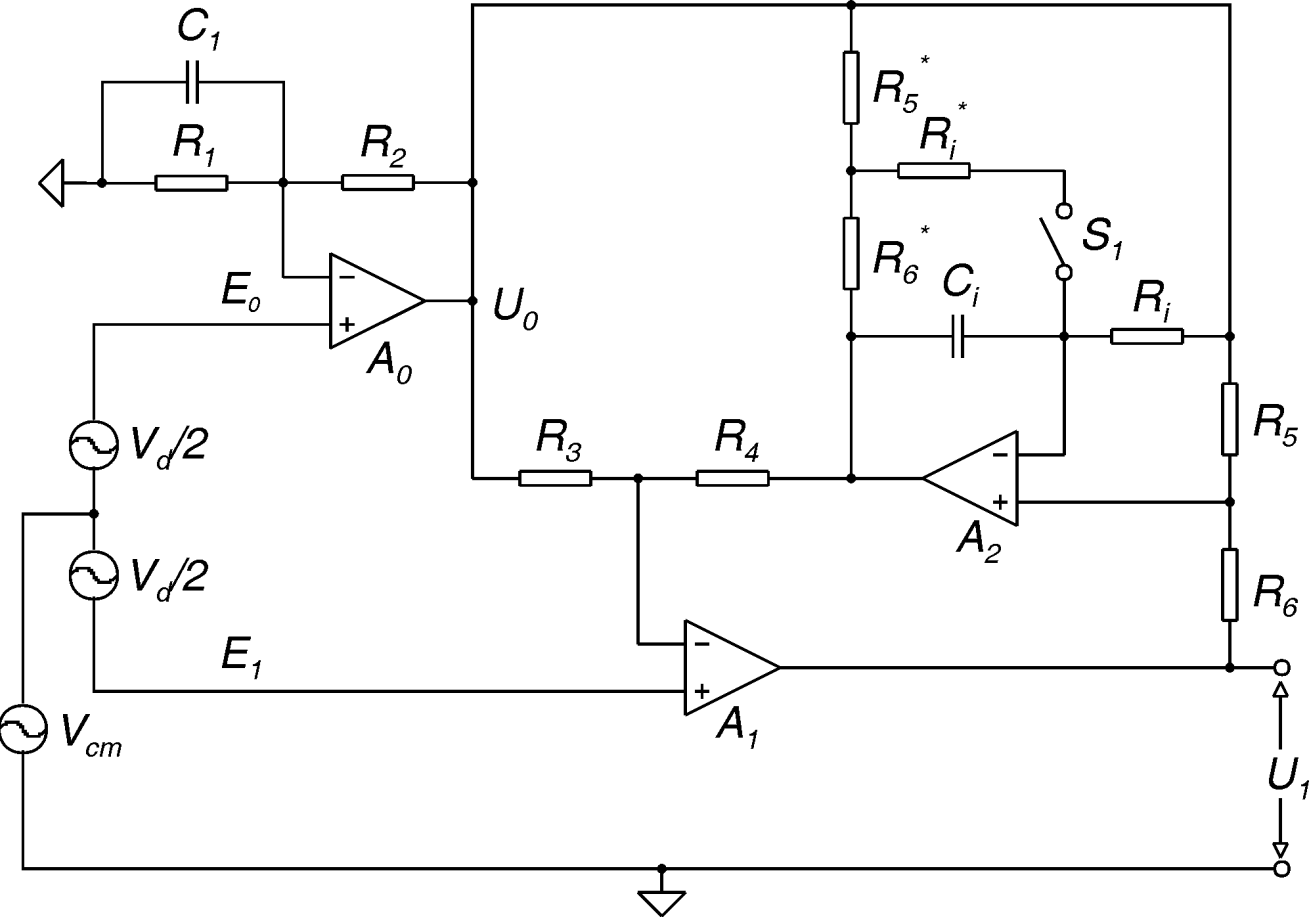
Schematic of the biopotential amplifier with active DC rejection/suppression.

**Figure 4:**
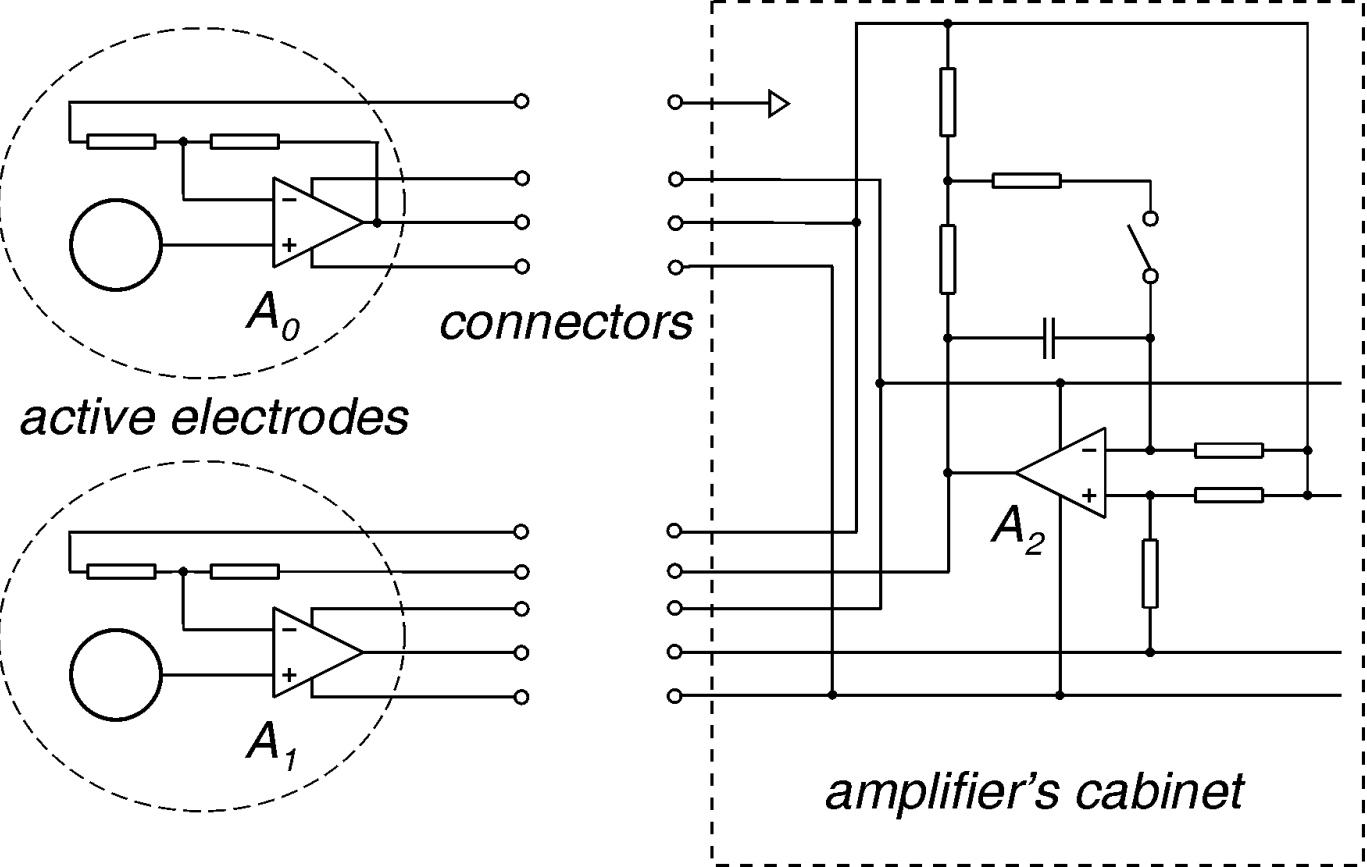
Simplified schematic of the biopotential amplifier with active electrodes.

**Figure 5:**
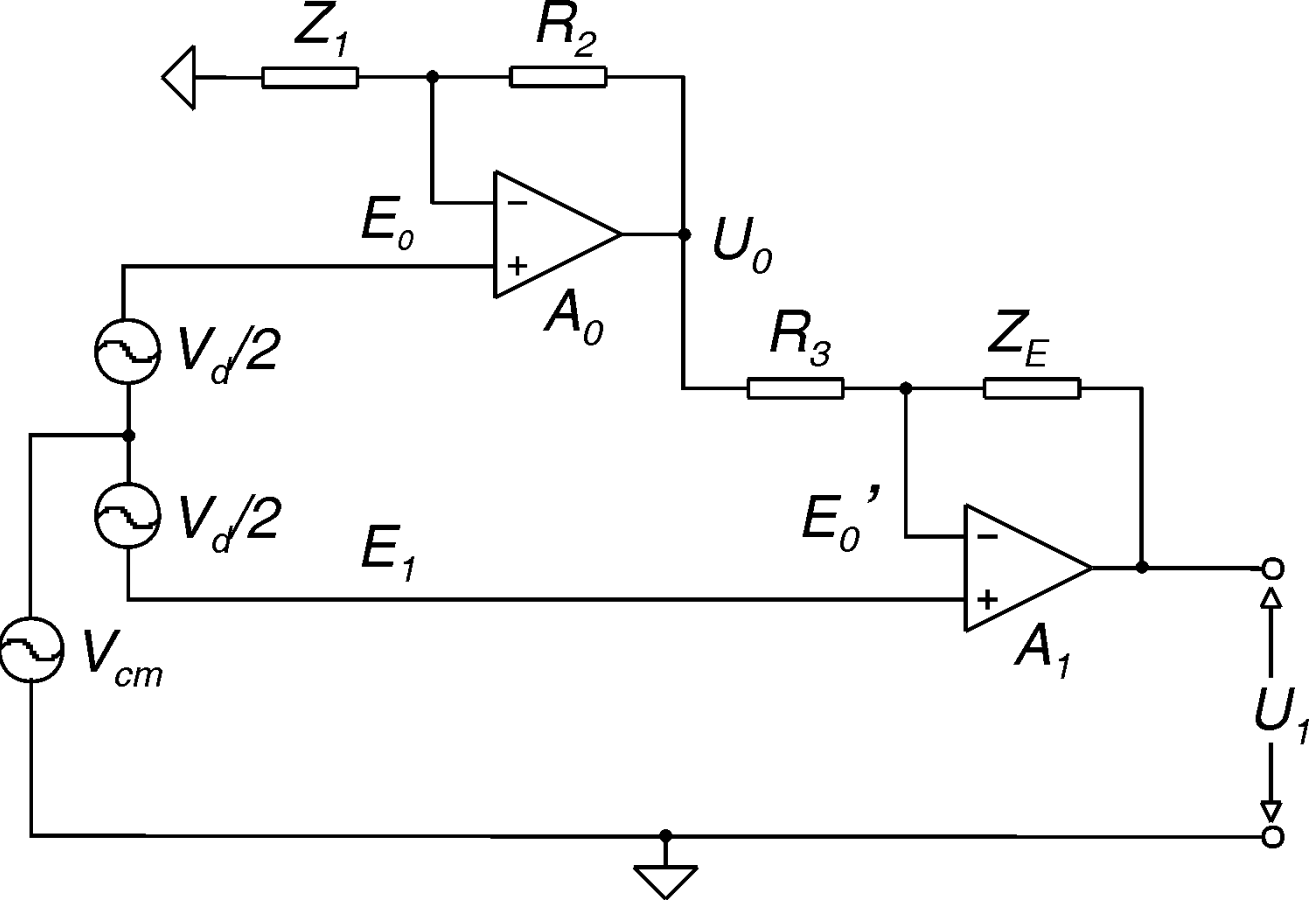
Schematic of the basic two-op-amp instrumentation amplifier.



Electrodes with impedance matching at the sensing site are referred to as active electrodes and have been designed since 1960's [15-17]. The electronic part of these transducers mostly consists of a buffer amplifier, but some have been designed to need only two lead connection wire [18,19]. However, as the signal is not amplified, buffers introduce significant noise and a low noise amplifier is still needed at the front-end. In order to avoid this drawback we used a two-op-amp biopotential amplifier [20] shown in Fig. 3, where op-amps A_0 _and A_1 _were integrated at the electrodes (Fig. 4), instead of using extra buffers. This resulted in lower noise and less parts, at the expense of increased number of electrode leads. The amplifier is based on the two-op-amp instrumentation amplifier shown in Fig. 5. The output voltage of the basic two-op-amp amplifier is

where A_d1_(s) is the differential-mode (DM) gain and A_c1_(s) is the common-mode (CM) gain of op-amp A_1_. If we take the usual definitions for the DM input signal, V_d_=(E_1_-E_0_), and for the CM input signal, V_c_=(E_1 _+ E_0_)/2, then the output voltage can be also written as

*U*_1_(*s*) = *A*_*d*_(*s*)*V*_*d *_+ *A*_*c*_(*s*)*V*_*c *_    (3)

It can be shown that the respective expressions for the DM gain A_d_(s), and the CM gain A_c_(s), are given by





where , τ_0 _and τ_1 _are the respective DM open loop gains, and the time constants of the first poles of op-amps A_0 _and A_1 _(assumed to be internally compensated), and A_c1_(s) is the CM gain of op-amp A_1_. It should be noted that the CM gain of op-amp A_0 _is omitted from (4) and (5), since its influence on both gains is insignificant.

Considering (4) and (5), then the common-mode rejection ratio CMRR(s) is



Assuming op-amps A_0 _and A_1 _are ideal then the only factor contributing to the CMRR is the mismatching of the resistors. Thus we can define a common-mode rejection ratio for the resistors, CMRR_R_. By taking 1/A_d0_(s) = A_c_(s) = 0 in (6) we obtain



Therefore CMRR_R_(s) approaches infinity if the relevant impedances are chosen according to



If the condition in (8) is fulfilled and op-amps A_0 _and A_1 _are ideal, then (4) simplifies to



Considering (7), equation (6) can be written as



where CMRR_A1_(s) is the CMRR of op-amp A_1_. Further if we assume that Z_1_, R_2_, R_3 _and Z_E _have tolerance t, then from (7) and (8) we can deduce that the worst case condition will be when Z_1 _= Z_10_(1-t), R_2 _= R_20_(1+t), R_3 _= R_30_(1-t) and Z_E _= Z_E0_(1+t) where Z_10_, R_20_, R_30_, and Z_E0_, are the respective nominal values. Equation (7) then can be written as



where . This means that large differential gain is desirable since very small tolerance components are expensive. Therefore, considering (11), equation (10) can be written as



The CMRR_A1 _has the form



where ω_r _is the frequency where CMRR_A1 _has decreased by 3 dB and is usually between 100 Hz and 1 kHz. The open loop gain A_d0_(s), also decreases at higher frequencies with a corner frequency ω_0 _= 1/τ_0 _which is usually lower than ω_r_, if A_0 _and A_1 _are of the same type. Therefore the CMRR is mainly determent at low frequency by the matching of the resistors and the DM gain, and at high frequencies by the open loop response of op-amp A_0_, rather than its CMRR. If we take the advantage of the fact implicit in (10), and achieve



then theoretically the CMRR becomes infinite. In part this can be achieved by the use of a capacitor and resistor in parallel for the impedance Z_1 _(Fig. 3), and then trimming R_2_. Thus the need of low-tolerance components is eliminated. Therefore Z_1_(s) will have the form



It has been shown [20] that a good approximation for the optimal value of the capacitor C_1 _is



where GBP_A0 _is the gain bandwidth product of op-am A_0_. Trimming R_2 _is a good solution for achieving an ultra high CMRR for demanding application, however it is not practical since the trimmer has to be incorporated in the electrode. Alternatively, Z_E _or R_3 _can be trimmed, which however will alter also the amplifier DM gain. Considering equation (12) it can be shown that for application with relatively high DM gain and proper op-amp selection, both trimming and compensation (C_1_) can be omitted, without significantly degrading the CMRR. For example, if the usual 1% tolerance resistors are used and op-amps with CMRR of 100 dB and DM open loop gain of 120 dB at 50 Hz, then for an amplifier with DM gain of 5000, a CMRR of 96 dB can be achieved without trimming.

In the amplifier circuit shown in Fig. 3, Z_E _is replaced with an active DC rejection/suppression circuit [20]. It includes an integrator (A_2_, R_i_, C_i_) and two potential dividers (R_6_, R_5 _and R_4_, R_3_). The amplifier can operate in AC-mode or in semi-AC-mode. The two modes are selectable by the switch S_1_: AC-mode with S_1 _open and semi-AC-mode with S_1 _closed. In AC-mode the DC signals are rejected, where in semi-AC-mode they are suppressed. If R_6 _= R_6 _*, R_5 _= R_5 _* and R_i _= R_i _*, then the respective expressions for the equivalent impedance Z_E_(s) for the two modes are given by





where τ_i _= R_i_C_i _is the time constant of the integrator,  and τ_2 _are the respectively the DM open loop gain and the time constant of the first pole of op-amp A_2_. Whenever R_i_>>R_5 _then *k *≈ (R_6_/R_5_+1), which is true with the time constants and voltage gains, typical in biopotential recordings. For signals bellow the amplifier high-pass cut-off frequency, Z_E_(s) decreases due to the active DC rejection/suppression circuit. For DC signals equation (8) is maximally imbalanced and thus CMRR_R_(0) ≈ A_d_(0). Since for biopotential amplifiers A_d_(0) is much lower than CMRR_A1_(0) and A_d0_(0), therefore CMRR(0) ≈ A_d_(0), which represents the worst case.

If we consider only the -3 dB bandwidth and assume that op-amp A_2 _is ideal, then (17) and (18) simplify to



Therefore, in this case (9) can be written as



which represents the mid band DM gain for both modes. After substituting (17) and (18) in (9), it can be shown that the respective DC differential gains for the two modes are given by



where 2*k *is approximately the DC gain of the stopped integrator (A_2_, R_i_, C_i_, R_i _*, R_5 _*, R_6 _*) in semi-AC-mode. Thus DC signals meet lower gain, in order to prevent saturation from large electrode offsets or other high DC potentials.

The active electrodes' input resistances R_iE0 _and R_iE1_, are not equal due to the different closed loop gains of op-amps A_0 _and A_1_, and can be expressed as



where R_iA0 _and R_iA1 _are the input resistances of op-amps A_0 _and A_1_. However, at higher frequencies, the electrodes' input impedances are much lower and about the same (assumed that A_0 _and A_1 _are of the same type), due to the op-amps' input and additional stray capacitance, being in parallel to the high op-amps' input resistance.

The output noise spectral density for the -3 dB bandwidth is approximately the same for both modes and can be written as



where e_n0_, e_n1 _and e_n2 _are the respective voltage noises of op-amps A_0_, A_1 _and A_2_.

Assuming E_0 _is connected to common (Fig. 3), then the amplifier transfer function H(s) is given by



After substituting Z_E_(s) and A_d1_(s) in (24), it can be shown that H(s) has three poles and two zeros for both modes. However, with the time constants and voltage gain used in the current application, one pole almost coincides with one zero. Therefore, H(s) can be approximated very well by a transfer function with two poles and one zero. The respective approximations for AC-mode and semi-AC-mode are given by





The circuits described by the transfer functions H_AC_(s) and H_sAC_(s) are stable because all the poles are situated in the left half of the complex s-plane and there are no resonance effects as the poles are on the real s-axis.

### Practical amplifier circuit

**Figure 6:**
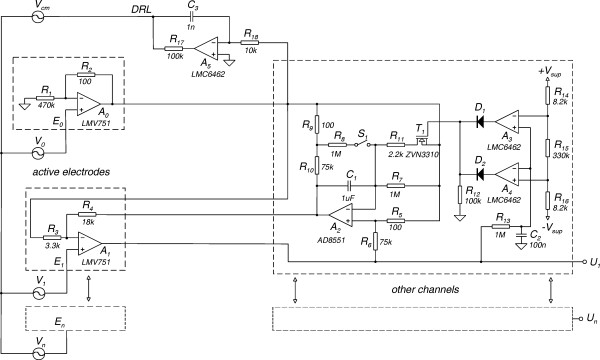
Multichannel biopotential amplifier with active electrodes and DRL circuit.

The schematic of a multichannel amplifier with active electrodes, built according to the design discussed is shown in Fig. 6. Each channel amplifies the signal between its input (E_1_...E_N_) and the reference input E_0 _(monopolar configuration). The output voltage of op-amp A_2 _is equal to the DC input voltage, multiplied by the ratio (R_3 _+ R_4_)/R_3 _for both modes. The choice of the resistor ratio (R_3 _+ R_4_)/R_3 _is a trade-off between DC input range and noise, since a low ratio enhances the noise contribution of op-amp A_2_. The ratio R_4_/R_3 _was chosen so that to allow a DC input voltage range of ± 370 mV, without saturating op-amp A_2_. The offset voltage at the amplifier output (U1) is the input offset voltage of op-amp A_2_, times the resistor ratio (R_5 _+ R_6_)/R_5_. In case of high DM gain, the output offset voltage would become unacceptably high. Thus, op-amp A_2 _was selected for its ultra-low offset voltage of 1 μV, low noise and high CM input range to prevent latch-up. Moreover A_2 _is a low input bias current type, which allows the use of high value input resistances without producing large offset voltages between its inputs. Op-amp A_0_, was selected with a relatively high GBP, in order to confine its influence on the CMRR at higher frequencies. Combining high DM gain, with high GBP op-amps, allowed Z_1 _to be implemented only with its active part R_1_, as thus the CMRR at 50 Hz was not significantly degraded. As shown in (23), the equivalent input noise is mainly determent by the noise of op-amps A_0 _and A_1. _They were implemented with the low-noise CMOS op-amp LMV751 in a SOT23-5 package.

Because of the large integrator's time constant, the amplifier has a very slow response after overloads (≤ 10τ_i_), caused by large signal disturbances. Thus a deblocking circuit was added at the cabinet's location, for temporary reduction of the time constant during overload [24]. It is controlled by the output voltage U1 through the low pass filter (R_13_, C_2_). The filter output controls two threshold triggers (A_3_, A_4_), which through D_1_,D_2 _control the MOS transistor T_1_, acting like a switch. When the output signal reaches its range limits (defined by R_14_, R_15_, R_16_), T_1 _opens and the new reduced time constant τ_i_* = (R_7_//R_11_)C_2_, pulls the output signal to the zero level. This state is maintained for additional hundred milliseconds (R_13_C_2_) and then is switched back to its original value.

The connection between the amplifier common and the signal source is implemented by a driven right-leg (DRL) circuit. The CM voltage at the output of A_0 _is reduced by a factor equal to the DRL circuit gain (A_DRL _= 314 at 50 Hz), which theoretically should give a 50 dB extra CMRR at 50 Hz. In addition, in case of a faulty op-amp, the DRL circuit will limit the maximum patient current to a safe level of 50 μA.

## Results

**Figure 7:**
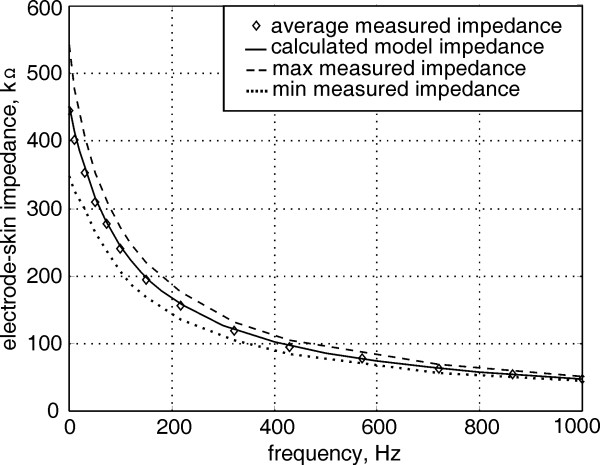
The average electrode-skin contact and the calculated model impedance against frequency. The plot shows also the minimum and the maximum data sets for five subjects.

The contact impedance of the proposed electrode, measured and averaged over five subjects, and its calculated model impedance are shown in Fig. 7. The values of the model elements were determent to give the closest agreement between the measured electrode-skin impedance and that of the model. The values are: R_s _= 300 Ω, R_d _= 450 kΩ and C_d _= 3 nF. The measuring technique is described by Bergey et al. [21] and was performed five minutes after the application of the electrodes to allow their impedance to settle to a constant value [13]. The electrodes were applied with moderate tension of 0.3 kg/cm^2 ^on an unprepared skin of the inner forearm. The impedance was lower when higher tension was applied or when sweat was present on the skin. The applied current density was 0.01 mA/cm^2 ^and no current density impedance dependence was observed [22]. The electrode-skin impedance showed two-decade spread between different subjects, which was also reported in other works [23].

**Figure 8:**
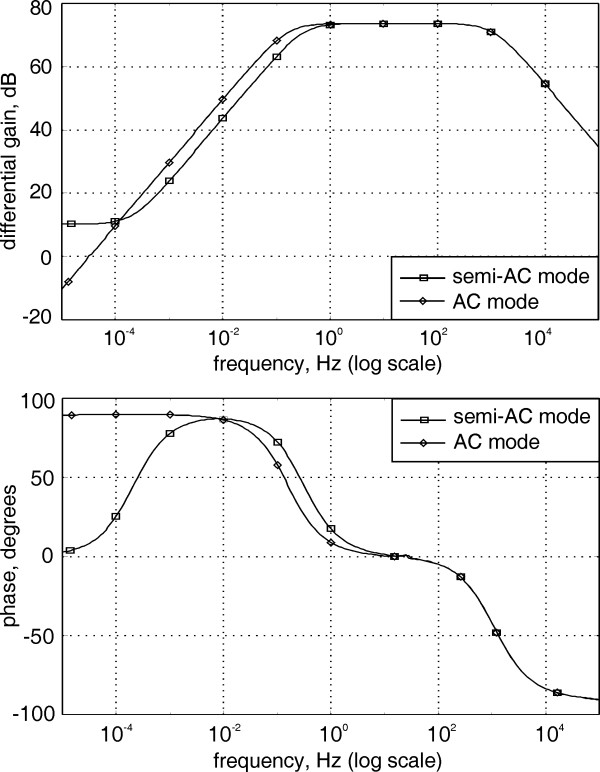
A plot of the amplifier frequency response.

Simulations of the amplifier circuit were carried out using PSPICE. The op-amps used in the model were with gain-bandwidth product (GBP) of 5 MHz, DM open loop gain of 120 dB and CMRR of 100 dB. The integrator time constant and the resistor ratios of the feedback loop were: τ_i _= R_i_C_i _= 1, R_4_/R_3 _= 6 and R_6_/R_5 _= 700. The amplifier frequency response plots, for both operating modes, are shown in Fig. 8. In semi-AC-mode the high-pass response is with 1^st ^order pole at 0.32 Hz and zero at 0.2 mHz. In AC-mode the high-pass response is with 1^st ^order pole at 0.16 Hz and zero at 0.16 μHz. The mid band DM gain is the same for both modes. Simulations were also carried out for the estimation of the power line interference due to induced displacement currents into the electrode leads. The increased stray capacitance between the power line and the amplifier, caused by the increased number of electrode leads, was taken into consideration in the simulation model. The results showed that the interference caused by the active electrode unshielded leads was insignificant.

Table 1 shows the amplifier specifications, measured with battery powered prototype and test equipment. All the parameters were in close agreement with those of the simulations. The peak-to-peak noise voltage measured at the amplifier output, with input terminals connected to common, was 10 mV_p-p_, or 2 μV_p-p _when referred to the input. The CMRR of the amplifier was 96 dB at 50 Hz, measured with imbalanced electrode impedances (ΔZ_e _= 47 kΩ). The maximum measured CMRR with DRL and a CM input signal of 4 V_p-p_, was 126 dB at 50 Hz, where the output signal level was approximately equal to the amplifier output voltage noise.

**Table 1:** Active electrode specifications

**Parameter**	**semi-AC-mode**	**AC-mode**
Bandwidth (-3 dB)	0.32–1000 Hz	0.16–1000 Hz
DC gain	3.22	≈ 0
AC mid band gain	74 dB	
Differential mode AC input range	0.005–1 mV_p-p_	
Differential mode DC input range	± 370 mV	
Common mode input range	± 2 V	
Input noise current	1 pA_rms _@ 0.1–200 Hz	
Input bias current	1.5 pA	
Input impedance, Active Electrode	320 MΩ @ 50 Hz (1000 GΩ //10 pF)	
CMRR	96 dB @ 50 Hz	
Output offset	0.7 mV	
Input noise voltage	2 μV_p-p _(0.33 μV_rms_) @ 0.1–200 Hz	
Power consumption	11 mW @ one channel	

**Figure 9:**
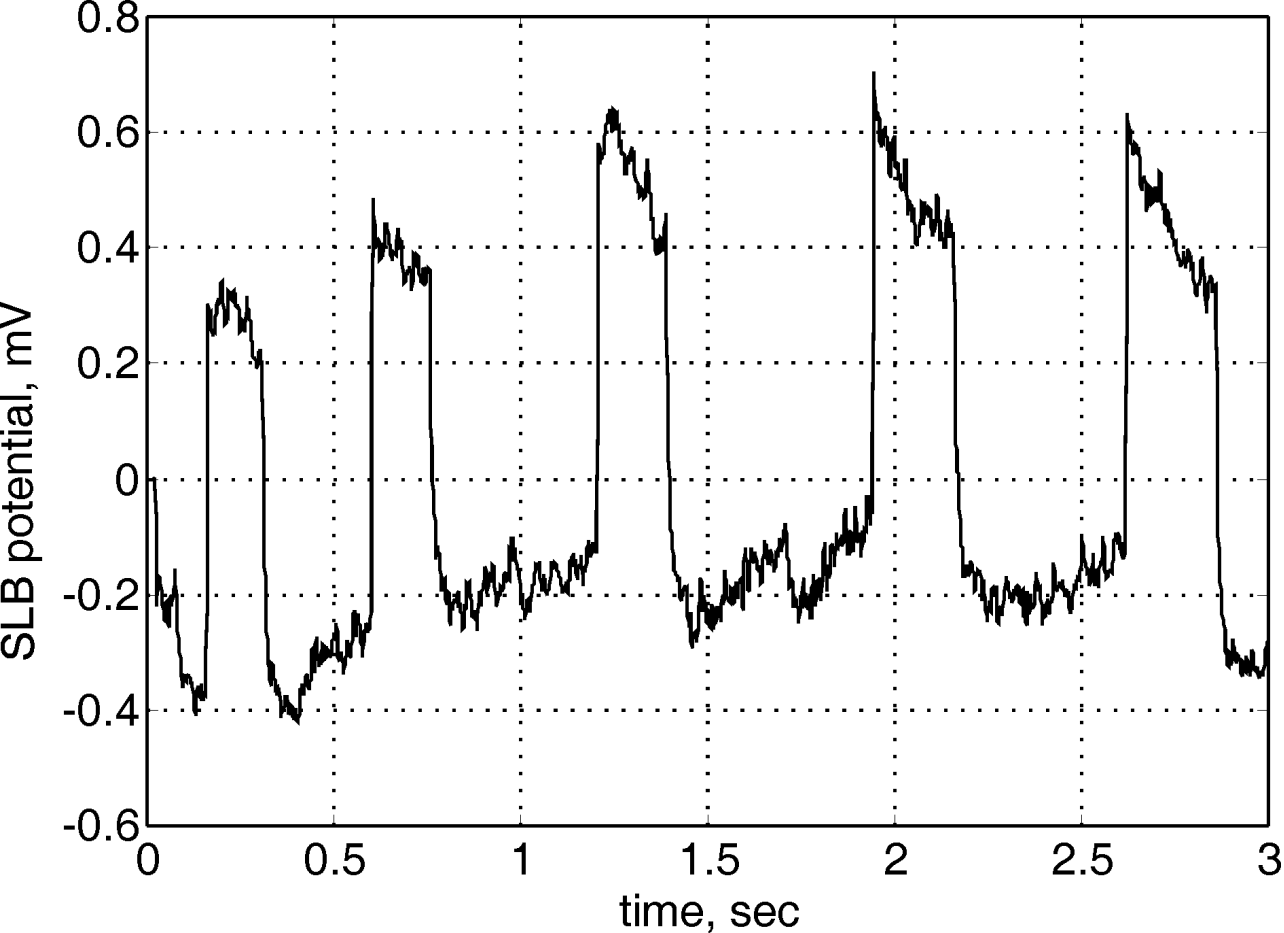
Biopotential acquired in semi-AC-mode from SLB TH-23 after low intensity laser stimulation.

A sample record from the practical application of the active electrode, obtained after a low intensity SLB laser stimulation, is shown in Fig. 9. The amplifier was battery powered and optically isolated by linear optocouplers. The bandwidth was limited to 200 Hz, by 6^th ^order low pass Bessel filter, and the signal was sampled with 1 kHz. The electrodes were connected with a high-density unshielded ribbon cable. One electrode (E_1_) was placed on SLB TH-23, near the eyebrow, where the reference (E_0_) was placed on the ear lobe. No electrolyte gel or skin preparation was applied. The measurements were performed in a typical laboratory room. All measurements showed almost complete absence of 50 Hz interference. The noise present in the signal is mainly compounded of artifacts from eye movements, electromyographic signals, and noise from the electrode-skin interface.

## Discussion

The best solution for an active electrode would be to perform the entire analog signal processing at the electrode site. This could be achieved with a custom made integrated circuit, but the cost would be much higher. We found a good alternative in using SMD technology and integrating only the front-end of the amplifier into the electrode.

The ultra high input resistance of the electrode is degraded at higher frequencies by the op-amp's input capacitance in parallel with the stray capacitance due to the electrode Printed Circuit Board (PCB). Nevertheless, combining an op-amp with low input capacitance and a proper PCB design, allowed a relatively high input impedance to be achieved at 50 Hz. That decreased the amplifier sensitivity to high electrode-skin impedance imbalances, by reducing the transformation of the CM interference signal into unwanted DM signal. Unfortunately, most data sheets do not properly specify op-amp's input capacitance, neither DM nor CM.

The active electrode presented is not suitable for applications requiring a low differential gain and large signal bandwidth due to the decreasing CMRR at higher frequencies, if not properly compensated. On the other hand, below the high-pass cut-off frequency, the CMRR is degraded by the active feedback circuit, and reaches its minimum value for DC signals, equal to the DM gain. The circuit can accept high value input filter resistances, which will also limit the patient auxiliary current in case of fault condition of op-amps A_0 _and A_1_. Because of the limited electrode space, it is preferable that the front-end op-amps feature internal electrostatic discharge protection circuitry, rather than building an external one.

## Conclusions

The new electrode anchoring system significantly reduced the electrode-skin impedance, its variation and motion artifact influences. The proposed amplifier fractionation resulted in lower noise and less parts. Moreover splitting the amplifier between the electrodes and the cabinet's location allowed the use of an automatic DC deblocking system and mode switching. The prototype tests showed that with the active electrode presented, SLB signals with relatively high quality could be recorded without skin preparation. The 50 Hz interference pickup by the electrode leads was practically eliminated. Because high electrode-skin impedances are tolerated, no electrolytic gel is needed. This allows fast application of the electrodes, minimizes patient discomfort and eliminates the risk of infection.

With proper op-amps selection, the active electrode specifications were found to be better or at least comparable to those of other existing designs. The design offers low noise and major reduction in parts, size and power consumption. It is currently used in studying laser provoked SLB potentials and their propagation, aiming to gain a better insight into the bio-stimulation effect of lasers in Medical Acupuncture.

## Authors' contributions

The authors contributed equally to this work
